# Regulatory Cytokine Expression and Preterm Birth: Case-Control Study Nested in a Cohort

**DOI:** 10.1371/journal.pone.0158380

**Published:** 2016-08-03

**Authors:** Thaís Basso de Brito Pereira, Erika Barbara Abreu Fonseca Thomaz, Flávia Raquel Fernandes do Nascimento, Ana Paula Silva de Azevedo dos Santos, Rosângela Lucena Fernandes Batista, Heloisa Bettiol, Ricardo de Carvalho Cavalli, Marco Antônio Barbieri, Antônio Augusto Moura da Silva

**Affiliations:** 1 Graduate Program in Public Health, Federal University of Maranhão, São Luís, Maranhão, Brazil; 2 Department of Public Health, Graduate Program in Public Health, Federal University of Maranhão, São Luís, Maranhão, Brazil; 3 Department of Pathology, Graduate Program in Health Sciences, Federal University of Maranhão, São Luís, Maranhão, Brazil; 4 Department of Puericulture and Pediatrics, Graduate Program in Child and Adolescent Health, São Paulo University, Ribeirão Preto, São Paulo, Brazil; 5 Department of Gynecology and Obstetrics, São Paulo University, Ribeirão Preto, São Paulo, Brazil; Hopital Robert Debre, FRANCE

## Abstract

**Background:**

Currently known risk factors explain only a small fraction of preterm birth (PTB). Previous PTB is one of the most important predictors. However, this information is not available in primiparous women. Few studies have looked at associations between regulatory cytokine expression (RCE) and PTB and the results are conflicting.

**Objective:**

To investigate the association of RCE–Interleukin 10 (IL-10) and Transforming Growth Factor β (TGF-β)–with PTB, and to assess whether bacterial vaginosis (BV) is involved in this relationship.

**Methods:**

This was a case-control study nested in a prospective cohort–called BRISA. Women with singleton pregnancies were interviewed from 22 to 25 weeks of gestational age (GA). Women were recruited from health services in São Luís, Brazil. A blood sample was collected and gynecological examination was performed. Serum IL-10 and TGF-β were determined using cytometric bead array. Nugent score >7 and/or the presence of clue cells were used for BV diagnosis. All PTB estimated by ultrasound dating performed before 20 weeks of gestational age were considered cases. Controls were selected by simple random sampling from the rest of the cohort, at a 2:1 ratio. Different models were tested, according to the main independent variable. Odds ratios (OR) and 95% confidence intervals (95%CI) were estimated by regression analyses.

**Results:**

The study included 327 pregnant women, 109 cases and 218 controls. No associations were found between BV and PTB (P = 1.44; 95%CI: 0.51–3.77). Low levels of IL-10 (OR = 2.92 95%CI: 1.38–6.16) or TGF-β (OR = 16.90 95%CI: 6.42–44.51) or both simultaneously (OR = 77.16 95%CI: 7.99–744.88) were associated with increasing odds of PTB, even after adjustment for confounding.

**Conclusion:**

Decreased RCE is a risk factor for PTB. This relationship, however, is not triggered by the presence of BV. Low IL-10/TGF-β levels from 22 to 25 weeks of GA could be used as early predictors of PTB. We suggest monitoring of these RCE, especially among primiparous women, for whom history of previous PTB is not available.

## Introduction

Previous preterm birth (PTB) is one of the most important predictor of further PTB [1,2]. However, this information is not available in primiparous women. Besides, currently known risk factors explain only a small fraction of PTB [1,3]. So, efforts should be done in order to identify markers of PTB, especially among primiparous women. Few studies have looked at associations between regulatory cytokine expression (RCE) and PTB and results are conflicting.

Among cytokine-producing immunological cells, T lymphocytes play an important role in the maintenance of pregnancy, especially in the balance between the T helper 1 (Th1) type immune response, predominantly proinflammatory, and the T helper 2 (Th2) response, predominantly anti-inflammatory [4]. Recently, however, it has been perceived that the maintenance of pregnancy depends not only on the Th1/Th2 ratio, but also on the action of T regulatory (Treg) cells [5]. The activation of these cells leads to the production of interleukin-10 (IL-10) and transforming growth factor β (TGF-β), which act by inhibiting the inflammatory response, especially the cytotoxic type. This may promote a balance between the pro- and anti-inflammatory immune responses [6].

During pregnancy, this homeostasis with a prevalent Th2 response may be impaired by infectious diseases [7]. A mechanism suggested for this possible association may be related to the action of cytokines via bacterial vaginosis–BV [8], which may increase the concentration of interleukins in the vagina and amniotic fluid [3]. This evidence suggests a mechanism of action mediated by proinflammatory cytokines with the induction of prostaglandin release, increasing uterine contractility and favoring premature rupture of the fetal membranes and PTB [9]. Another possibility is that BV may imbalance the regulatory cytokine expression and thereby lead to PTB. However, evidences are controversial.

Low TGF-β levels but high IL-10 have been found in blood from the umbilical cord of pregnant women with placental inflammation [10]. Some authors found low IL-10 [11,12] levels in blood from the umbilical cord of preterm neonates, but others have found high IL-10 [1] in the plasma of pregnant women who experienced PTB. Higher levels of TGF-β were associated with increased odds of PTB<35 weeks [4]. However, in other study this association was not spotted [1].

Few longitudinal studies have been conducted in humans with large samples, appropriate classification of gestational age (GA) and of BV, with adjustment for confounding factors. Besides, most studies that have evaluated the possible role of cytokines in PTB used specimens from the placenta or umbilical cord, which are not practical in routine medical practice. Thus, in the present study we tested the hypothesis that PTB is regulated by IL-10 and TGF-β cytokines, measured in the peripheral blood, and that BV impairs this regulation.

## Materials and Methods

A case-control study nested in a prospective cohort (BRISA–Brazilian Ribeirão Preto and São Luís birth cohort study) [13] was carried out according to STROBE guidelines.

In the base cohort, pregnant women were recruited at four health services in São Luís, Brazil, during a prenatal visit, representing a convenience sample. Only women with a single fetus and with GA measured by obstetrical ultrasound (US) at less than 20 weeks of pregnancy were included (n = 1,447). Recruitment lasted from February/2010 to February/2011. The women were interviewed at 22–25 weeks of GA and 20mL blood was collected by venipuncture. Blood was centrifuged for serum separation and serum was placed in Eppendorf tubes, labeled and stored in a freezer at -80°C.

Ninety-five percent of these women were re-interviewed at childbirth (n = 1,387). All patients whose babies were born preterm were included in the case-control study and defined as cases. The controls were selected from the rest of the cohort by simple random sampling. This strategy reduces costs but keeps the statistical power.

It was estimated that a sample of 264 women (88 cases and 176 controls) would have a 90% power to identify significant Odds Ratios (OR) for associations between cytokine levels and BV with PTB equal to or higher than 2.5, considering a 50% frequency of exposure, control/case ratio of 2 and a 95% confidence interval (95%CI).

PTB, defined as birth at less than 37 weeks of gestation according to the US was the dependent variable. PTB was classified as medically indicated, iatrogenic or spontaneous. PTB was considered medically indicated when a caesarean section was performed due to fetal distress (reduced heart rate of the fetus / or presence of meconium in the womb), cephalopelvic presentation (baby sitting / wrong position) or dead; or in case of maternal hemorrhage, pre-eclampsia or eclampsia. PTB in the presence of elective caesarean section was considered iatrogenic. When PTB occurred under normal delivery, this was considered spontaneous. Type of PTB was confirmed based on data from the medical notes. Among children examined at the base cohort (n = 1,447), all who were born preterm (n = 109) were considered cases.

Main independent variables were BV and serum concentrations of RC (IL-10 and TGF-β). For the diagnosis of BV, the women were submitted to gynecological examination with the use of a disposable speculum. The criteria for the diagnosis of BV were the Nugent, Krohn and Hillier [14] score and/or the presence of clue cells. The latter criterion is based on Gram staining of a vaginal smear and count of the existing morphotypes of *Lactobacillus*, *Gardnerellas*/*Bacteroides* and *Mobiluncus*. The morphotypes were scored from 1+ to 4+ according to the number of microorganisms per field and summed. The presence of a score ≥7 was considered to indicate BV [14].

The cytometric bead array (CBA) assay was used for serum cytokine determination. All reagents used were from the kit for human cytokines Th1/Th2/Th17 purchased from Becton Dickinson Biosciences (San Jose, CA, USA). The cytometer was calibrated according to manufacturer recommendations. After a reading of the standard curve and of the sample curve, the data were analyzed with the FCAP Array Software (Becton Dickinson, San Jose, CA, USA) and the values are reported as pg/mL.

The following covariables, obtained by direct interview with the women, were considered: a) Sociodemographics–age (in years); self-reported race/color, classified as white or other (including black, brown/mulatto or yellow/oriental); marital status (with or without a partner); years of study (up to 8 or ≥9 years) and economic class, categorized according to the criteria of the Brazilian Association of Research Enterprises [15], as: A-B (more affluent), C or D-E. This classification is based on the number of color television, radio, bathroom, automobile, salaried maid, washing machine, DVD, refrigerator and freezer present in the home; as well as on the education of the household head. Each answer receives a certain score and total score ranges from 0 to 46. Classes are categorized into A (35–46 points), B (23–34 points), C (14–22 points), D (8–13 points) or E (0–7). b) Reproductive health–number of pregnancies (primigesta or multigesta); and a previous history of PTB (yes or no); c) Morbidity during the current pregnancy–hypertension, including both chronic hypertension preexisting pregnancy or preeclampsia in pregnancy (yes or no); d) Maternal life habits during the current pregnancy–consumption of alcoholic drinks (yes or no); smoking (active, passive or no exposure); and use of illicit drugs, according to a self-applied questionnaire (yes or no).

Most data were obtained during the prenatal period. Only variables such as drug use, hypertension during pregnancy and PTB were obtained at childbirth.

A descriptive analysis of the data was performed first. The Shapiro-Wilk test and graphic analysis (box-plots and histograms) were adopted in order to assess data distribution. The nonparametric Mann-Whitney was used to estimate differences in median IL-10 and TGF-β levels between cases and controls. Correlation between TGF-β and IL-10 was analyzed by Spearman correlation test.

The associations of BV and of IL-10 and TGF-β levels with the outcome (PTB) were determined by means of the OR and 95%CI in logistic regression analyses. For the adjustment of confounders, variables with a P-value <0.20 were selected if associated with the outcome in the non-adjusted model. Variables with a P-value <0.10 were left in the adjusted model. For regression analysis, the serum levels of the cytokines were dichotomized according to the median values. Four different models were constructed, considering PTB as the outcome. Model I tested the association between BV and PTB. Model II tested the association between low IL-10 levels and PTB. Model III, the association between low TGF-β levels and PTB. Model IV considered IL-10 and TGF-β jointly, categorized as: IL-10 and TGF-β above the median (reference), only IL-10 below the median, only TGF-β below the median, and both variables below the median.

Muticolinearity was tested in each model using the _rmdcoll command of Stata. Interactions between low BV and low cytokines levels were explored using the likelihood ratio test. The Stata 11.0 software (Stata Corp., College Station, TX, USA) was used for statistical analysis (Alpha = 0.05).

Missing data were excluded from analysis. Additionally, sensitivity analysis [16] was performed to estimate the effect of missing data on the associations, using the worst-case scenario strategy. The associations between cytokine levels and PTB were estimated considering two extreme situations. Firstly, values higher than the median were imputed for all missing cytokine data and secondly, values lower than the median were imputed. The results would respectively represent the highest and lowest values that the measures of association could have assumed.

The Institutional Review Board of the Federal University of Maranhão approved the study (Process: 4771/2008-30) in April 8, 2009. All subjects gave written informed consent to participate.

## Results

The study was conducted on 327 pregnant women, 109 cases and 218 controls. Of the total cases, 83.5% (n = 91) are late PTB and 16.5% (n = 18), early. Most PTB was considered spontaneous (n = 44, 40.7%), followed by iatrogenic (n = 41, 37.1%) or medically indicated (n = 24, 22.2%). The mean age of the pregnant women was 25.9 (±5.7) years, with 80.7% of them being 20–34 years old. Sixty-seven percent of the pregnant women belonged to economic class “C” with 57 of them (17.9%) having a *per capita* income of less than one minimum wage (U$ 306.12 monthly). Only 14.1% had more than eight years of study.

The PTB rate was higher among women aged ≥35 years than among younger women (P = 0.013), among women with a previous history of PTB (P<0.001), with gestational hypertension (P = 0.001) and those who reported use of illicit drugs (P = 0.017) (Table 1).

**Table 1:** Characterization of the study population divided into case (n = 109) and control (n = 218) groups. São Luís, MA, Brasil. 2010–2012.

Variables	n	Control (%)	Case (%)	P-value
Age (years)				0.013^d^
<20	38	68.4	31.6	
20–34	264	68.9	31.1	
≥ 35	25	40.0	60.0	
Race/Skin colour				0.809^d^
White	58	65.5	34.5	
Not white^a^	268	67.2	32.8	
Schooling (years)				0.216^d^
≥ 9	46	58.7	41.3	
Up to 8	281	68.0	32.0	
Economic class^b^				0.079^d^
A-B	63	58.7	41.3	
C	220	66.4	33.6	
D-E	44	79.5	20.5	
Marital status				0.7616^d^
With a partner	266	67.3	32.7	
Without a partner	61	63.9	36.1	
Number of pregnancies				0.820^d^
Primigesta	145	66.2	33.8	
Multigesta	181	67.4	32.6	
Missing	1			
Previous preterm delivery				<0.001^d^
No	259	80.7	19.3	
Yes (1 or more)	65	10.8	89.2	
Missing	3			
Hypertension during pregnancy				0.001^d^
No	252	71.8	28.2	
Yes	73	50.7	49.3	
Missing	2			
Elective cesarian section				<0.001^d^
No	226	74.8	25.2	
Yes	100	49.0	51.0	
Missing	1			
Bacterial vaginosis during pregnancy^c^				0.521^d^
No	232	67.7	32.3	
Yes	40	62.5	37.5	
Missing	55			
Smoking during pregnancy				0.924^e^
No	274	66.8	33.2	
Only passive	43	65.1	34.9	
Active	8	75.0	25.0	
Missing	2			
Alcohol consumption during pregnancy				
No	298	66.8	33.2	0.891^d^
Yes	29	65.5	34.5	
Use of illicit drugs during pregnancy				
No	320	66.5	32.5	0.017^e^
Yes	6	16.7	83.3	
Missing	1			

DOI: 10.6084/m9.figshare.3473510

^a^Includes black, brown/mulatto, yellow/oriental.

^b^Economic class according to ABEP (Brazilian acronym for the Brazilian Association of Research Enterprises) 2009 criteria.

^c^Using the Nugent criterion.

^d^Chi-square test.

^e^Fisher exact test.

IL-10 and TGF-β levels did not show normal distribution (P<0.001). There was a great variability in the levels of these regulatory cytokines. Median levels of serum IL-10 (P = 0.003) and TGF-β (P<0.001) were higher in the control group (Table 2). There was no significant correlation between TGF-β and IL-10 (R = 0.02; p = 0.731).

**Table 2:** Mean and median levels of regulatory T cytokines (IL-10 and TGF-β) in case and control groups. São Luís, MA, Brazil. 2010–2012.

Cytokine (pg/mL)	Total			Term birth			Preterm birth			P-Value^a^
	n	Mean (SD)	Median (Q1-Q3)	n	Mean (SD)	Median (Q1-Q3)	n	Mean (SD)	Median (Q1-Q3)	
IL-10	282	0.43 (1.07)	0.0 (0.0–0.24)	190	0.54 (1.24)	0.0 (0.0–0.48)	92	0.19 (0.49)	0.0 (0.0–0.075)	0.003
TGF-β	281	934,088.4 (3,562,447.0)	174,690 (46,338–423,007.0)	194	1,288,991.0 (4,235,398.0)	297,294.0 (70,704.0–523,408.0)	87	142,696.0 (379,946.5)	65,903.0 (23,979.0–111,170.0)	<0.001

SD: standard deviation; M: median; Q1-Q3: interquartile range. DOI: 10.6084/m9.figshare.3473543

^**a**^Mann-Whitney test.

No associations were found between BV and PTB, even after adjustment (Table 3). The adjusted model explained 36.4% of the variability in the occurrence of PTB (R^2^ = 0.364) (Model I). As BV was not associated with the outcome, the effect of mediation of BV in the association between RC and PTB was not tested. In addition, there was also no interaction between BV and cytokines (LR-test p-value > 0.05).

**Table 3:** Association of bacterial vaginosis, IL-10 and TGF-β with preterm birth adjusted for confounding variables. São Luís, MA, Brazil. 2010.

Model	Main independent variable (n)	Non-adjusted association			Adjusted association		
		OR	95%CI	P	OR	95%CI	P
I	Vaginosis						
	Yes (40)	1.25	0.62–2.52	0.521	1.44	0.51–3.77	0.457^a^
	No (232)	1.00			1.00		
II	IL-10 (pg/mL)						
	≤ 0.01 (172)	2.17	1.26–3.73	0.005	2.92	1.38–6.16	0.005^b^
	≥ 0.01 (110)	1.00			1.00		
III	TGF-β (pg/mL)						
	0–174,690 (140)	14.03	6.97–28.25	<0.001	16.90	6.42–44.51	<0.001^b^
	> 174,690 (141)	1.00			1.00		
IV	T regulatory cytokines (pg/mL)						
	IL-10 ≤ 0.01 and TGF-β ≤ 174,690 (81)	94.64	12.41–721.86	<0.001	77.16	7.99–744.88	<0.001^b^
	IL-10 > 0.01 and TGF-β ≤ 174,690 (57)	33.82	4.36–262.46	0.001	19.09	1.92–189.73	0.012 ^b^
	IL-10 ≤ 0.01 and TGF-β > 174,690 (87)	6.49	0.81–52.30	0.079	3.48	0.32–38.12	0.307 ^b^
	IL-10 > 0.01 and TGF-β > 174,690 (51)	1.00			1.00		

OR = odds ratio. 95%CI = 95% confidence interval. DOI: 10.6084/m9.figshare.3473546

^a^Estimate adjusted for subject’s age, economic class, hypertension during pregnancy, use of illicit drugs during pregnancy and previous preterm delivery.

^b^Estimates adjusted for subject’s age, hypertension during pregnancy, use of illicit drugs during pregnancy, elective cesarean section and previous preterm delivery.

IL-10 levels below the median increased the chance of occurrence of PTB in bivariate analysis. This association persists even after adjustment for confounding variables. PTB was 192% (OR = 2.92) higher among women with IL-10 levels ≤ 0.01 (median value) compared to women with higher levels (Model II). Women with low TGF-β levels had a greater chance of occurrence of PTB in both simple and multiple regression analysis (Model III) (Table 3). The adjusted models explained 34.4% (Model II) and 46.5% (Model III) of the variability in the occurrence of PTB.

Model IV revealed that low levels of IL-10 alone were not associated with an increased chance of PTB. However, low levels of TGF-β alone led to 19.09 greater chance of giving birth preterm compared to women with higher IL-10 and TGF-β levels. In addition, after adjustment for confounders, when both IL-10 and TGF-β concentrations were low, the chance of PTB increased 77.16 times (R^2^ = 0.497) (Table 3). No multicolinearity was detected in any of the adjusted models.

Among these women, BV could not be determined in 19 cases (17.4%) and in 36 controls (16.5%), TGF-β could not be determined in 22 cases (20.2%) and 24 controls (11.0%) and IL-10 in 17 cases (15.6%) and 28 controls (12.8%). IL-10 missings were greater among white mothers compared to all others (22.4% *vs* 11.9%; P = 0.036) and among those who did not live with a partner (22.9% *vs* 11.6%; P = 0.021), although without a statistical significant difference between cases and controls. However, TGF-β missings were greater among mothers of PTB infants (20.2% *vs* 11.0%; P = 0.024), among white mothers (24.1% *vs* 11.9%; P = 0.016), among those belonging to the A-B (14.3%) and C (16.4%) economic classes compared to those belonging to the D-E (2.3%) class (P = 0.031), and among those who did not live with a partner (23.0% *vs* 12.0%; P = 0.027).

Sensitivity analysis was performed to test if associations between TGF-β and PTB might have been due to the differential missings between cases and controls during follow-up. These evaluations are not shown for IL-10 because no difference in explanatory variables between performed and missing exams was significant. Considering the values of all missing TGF-β data to be below the median, the estimates continued to be similar to those detected in the present study both for non-adjusted (OR = 13.16) and adjusted (OR = 18.35) analysis. When values above the median were attributed to all missing values, the non-adjusted (OR = 5.54) and adjusted (OR = 5.58) odds ratios continued to be significant.

## Discussion

### Principal findings

Low IL-10 and TGF-β levels were risk factors for PTB. These changes, however, were not influenced by BV. This is the first report of associations between RCE in the plasma of healthy pregnant women in a nested case-control study conducted in Brazil, in which GA was estimated by US and cytokines were determined before PTB. This is also the first study to assess the joint associations of both regulatory cytokines with the occurrence of PTB.

A few studies have related PTB with IL-10 and TGF-β levels [1,3,4,10–12,17–19]. Some of these studies assessed spontaneous abortion in an animal [18] or human [19] model, and others determined the cytokines in the umbilical cord [10,11] or amniotic fluid [3], thus differing from the methodology employed in the present investigation, in which these regulatory cytokines were measured in the serum. Some involved a small sample size [1,17] and others investigated factors associated with PTB<35 weeks of GA [4,20] or included only pregnant women with a previous history of PTB [20]. Another difference was the time of cytokine determination, which ranged from 9–33 weeks of GA [1,4,12,17,20] or even occurred at birth [10,11].

Two studies have also identified an association between low serum IL-10 levels and increased odds of PTB in humans [12,20]. Recurrent PTB at less than 35 weeks of GA was associated with a lower production of IL-10 in the second trimester of pregnancy [20], suggesting that high IL-10 levels play a protective role against PTB. In contrast, some authors did not detect an association between serum IL-10 and PTB [4], while others [1,10,11] have reported that increased serum IL-10 levels are associated with increased odds of PTB, diverging from the results of the present investigation.

Both regulatory cytokines have never been studied jointly previously. We observed that when IL-10 levels are increased, but TGF-β levels are decreased, the risk of PTB is high, probably not because of the high IL-10, but due the low TGF-β concentrations. This is supported by our finding of a potential synergism between low levels of both cytokines, by increasing the chance of PTB.

A study of 142 pregnant women with a threat of spontaneous abortion [10] and a cohort study of 926 pregnant women [4] did not detect an association between PTB and serum TGF-β level. In contrast, using an umbilical cord sample, some studies [10,11] showed that increased TGF-β levels were associated with reduced odds of PTB, in agreement with the results of the present investigation. In these studies, cytokines were not measured at the same time of pregnancy.

Some studies [18,19] detected reduced serum IL-10 and TGF-β levels among pregnant women who aborted, providing more evidence of the importance of these cytokines for the maintenance of pregnancy.

Disagreements may be partly explained by methodological differences in study design, sample size, gestational period and biological samples for cytokine determination, criterion for GA classification, and in the variables considered for adjustment. It has been already demonstrated that serum cytokines show higher sensitivity and specificity than amniotic fluid and umbilical cord cytokines [20]. In addition, the use of these samples (collected only at birth) does not seem to have good practical applicability for the prevention of PTB.

BV was not found to be associated with the concentrations of regulatory cytokines during pregnancy or with PTB. The lack of an association between BV and PTB has been also demonstrated in previous studies [21,22], although no consensus has been established [8]. This may be explained by differences in the local immune responses and in genetic determinism [23], which were not investigated in these studies.

### Strengths and weaknesses

Although the data for this cohort are not population based, the important factor is to obtain appropriate numbers of women in each category of the variables under study in order to establish a contrast with adequate statistical power. In addition, the greater socioeconomic homogeneity of the sample represented an advantage because it reduces confounding. Besides, although cases and controls were not similar in relation to maternal age, hypertension, and drug use, these variables were considered in the adjusted analyses.

Despite missing data, BV and IL-10 missings did not differ between cases and controls. However, TGF-β missings were greater among cases (P = 0.024). However, analysis of sensitivity [16] showed that, even in the worst-case scenario, in case all the missing values for TGF-β were above the median, non-adjusted and adjusted associations would continue to be statistically significant. These observations support validity of the findings.

In case-control studies, it is difficult to ensure a correct sequence of events, however, the design nested in a prospective cohort reduces these errors. In the present study, US has been used as the gold standard for the estimation of GA, minimizing measurement bias [24]. Objective techniques were also adopted for the measurement of the principal exposures, with previous calibration of the instruments. Another advantage was the use of maternal serum, which is easily accessible and permits earlier identification of possible changes in cytokine levels.

### Plausibility and unanswered questions

It is still unclear whether the inflammatory response can actually trigger PTB [25,26]. The disequilibrium between the action of pro- and anti-inflammatory cytokines can play a role in the initiation of labor through different pathways–infection, decidual hemorrhage, uteroplacental ischemia, cervical disease, and/or immunological phenomena [26]. Current evidence indicates that the equilibrium between the pro- or anti-inflammatory response is mediated by the action of IL-10 and TGF-β [5]. Thus, these regulatory cytokines play an important role in the maintenance of pregnancy. Treatment with IL-10 for intra-amniotic infections in animal studies reduced uterine contractions [27]. In humans, serum IL-10 concentrations are higher in the first trimester of pregnancy and lower at term delivery and among nonpregnant controls, suggesting that a negative IL-10 regulation occurs as part of the inflammatory process necessary for term delivery [28].

Regulatory T lymphocytes and macrophages classically activated or alternatively activated in response to microbial products produce cytokines [29,30]. However, although it is recognized that these immune cells are functionally altered during pregnancy [31], little is known about the regulation of pro- and anti-inflammatory cytokines in response to an inflammatory stimulus during pregnancy.

Although it has been previously observed that infectious diseases may interfere with the predominantly Th2 immune response [7], in the present study BV was not found to be associated with the concentrations of regulatory cytokines during pregnancy or with PTB.

This is plausible, since BV examination and cytokine determination were performed once at the same time, another possible explanation for the lack of association may be that BV were not identified because it may have subdued shortly after the activation of the cascade of inflammatory mediators. PTB may be a life-course condition so it is possible that RC expression was impaired before pregnancy.

Decreased RC expression, as measured by IL-10 and TGF-β, is a potential risk factor for PTB. This relationship, however, was not triggered by BV measured from 22 to 25 weeks of pregnancy. Serum IL-10 and TGF-β levels from 22 to 25 weeks of GA may be markers of high clinical value for the identification of groups at risk for PTB, especially among primigravidae. Further research should also focus on early life factors and not only on risk factors during pregnancy.
